# Supplementary material for: Promoting youth mental health during the COVID-19 pandemic: A longitudinal study
Source: PLoS One. 2021 Aug 11;16(8):e0255294. doi: 10.1371/journal.pone.0255294 (PMC8357139; doi:10.1371/journal.pone.0255294)
Supplement: S1 File — (DOCX) [file pone.0255294.s001.docx]

**S1 File**

**S1 Table. Frequency of exposure to pandemic-related stressors (by domain).**

| **Pandemic-Related Stressors** | | | |
| --- | --- | --- | --- |
| **Stressor Domain** | **Number of Stressors** | **Frequency (%) at T1** | **Frequency (%) at T2** |
| Health | 0 | 107 (48.2%) | 74 (40.2%) |
|  | 1 | 71 (32.0%) | 57 (31.0%) |
|  | 2 | 33 (14.9%) | 36 (19.6%) |
|  | 3 | 8 (3.6%) | 12 (6.5%) |
|  | 4 | 6 (2.7%) | 4 (2.2%) |
|  | 5 | 0 (0%) | 1 (0.5%) |
|  | 6 | 0 (0%) | 0 (0%) |
|  | 7 | 0 (0%) | 0 (0%) |
| Financial | 0 | 145 (65.3%) | 133 (7.23%) |
|  | 1 | 55 (24.8%) | 35 (19.0%) |
|  | 2 | 19 (8.6%) | 15 (8.2%) |
|  | 3 | 6 (2.7%) | 1 (0.5%) |
|  | 4 | 0 (0%) | 0 (0%) |
| Social | 0 | 165 (74.3%) | 162 (88.0%) |
|  | 1 | 46 (20.7%) | 15 (8.2%) |
|  | 2 | 12 (5.4%) | 7 (3.8%) |
|  | 3 | 2 (0.9%) | 0 (0%) |
|  | 4 | 0 (0%) | 0 (0%) |
| School | 0 | 130 (58.6%) | 126 (68.5%) |
|  | 1 | 84 (37.8%) | 58 (31.5%) |
|  | 2 | 11 (5.0%) | 0 (0%) |
| Physical Environment | 0 | 202 (91.0%) | 159(86.4%) |
|  | 1 | 23 (10.4%) | 25 (13.6%) |

A composite of pandemic-related stressors at T1 was used in all analyses. We include the number of pandemic-related stressors at T2 to illustrate the ongoing nature of the pandemic at T2.

**S2 Table. Distribution of potential protective factors and psychopathology symptoms.**

| **Potential Protective Factor (continuous variables)** | **Mean (Standard Deviation)** |
| --- | --- |
| Physical Activity (mins / week) | *M* = 195.34 (159.09) |
| Time in Nature (days / week) | *M* = 2.02 (2.12) |
| Days Outdoors (days / week) | *M* = 2.97 (2.29) |
| Screen Time (hours) | *M* = 7.288 (4.95) |
| Routine (continuous scale 1-4) | *M* = 1.59 (0.87) |
| **Potential Protective Factor (binary variables)** | **Percentage of Participants** |
| Consuming Less than 2 hours of News per Day | 66.67% |
| Getting Recommended Amount of Sleep per Night | 50.9% |
| Adaptive Coping Strategies | 52.2% |
| Helping | 56.2% |
| **Psychopathology** | **Mean (Standard Deviation)** |
| Baseline Internalizing (CBCL) | *M* = 54.49 (9.89) |
| Baseline Externalizing (CBCL) | *M* = 52.05 (8.60) |
| Internalizing T1 (SDQ) | *M* = 5.35 (3.51) |
| Externalizing T1 (SDQ) | *M* = 6.95 (3.49) |
| Internalizing T2 (SDQ) | *M* = 5.51 (3.60) |
| Externalizing T2 (SDQ) | *M* = 7.20 (3.61) |

**S3 Table. Bivariate correlations between all study variables.**

|  | Stress | AGE | | SES | | INT Base | | EXT Base | | INT T1 | | EXT T1 | | INT T2 | | EXT T2 | | Phys | | Nature | | Outdoor | | Screen | | News | | Sleep | | Routine | | Coping | |  | |  |
| --- | --- | --- | --- | --- | --- | --- | --- | --- | --- | --- | --- | --- | --- | --- | --- | --- | --- | --- | --- | --- | --- | --- | --- | --- | --- | --- | --- | --- | --- | --- | --- | --- | --- | --- | --- | --- |
| Stress |  |  | |  | |  | |  | |  | |  | |  | |  | |  | |  | |  | |  | |  | |  | |  | |  | |  | |  |
| AGE | 0.003 | |  | |  | |  | |  | |  | |  | |  | |  | |  | |  | |  | |  | |  | |  | |  | |  | |  | |
| SES | -0.161* | | 0.037 | |  | |  | |  | |  | |  | |  | |  | |  | |  | |  | |  | |  | |  | |  | |  | |  | |
| INT Base | 0.165* | | 0.341** | | -0.184* | |  | |  | |  | |  | |  | |  | |  | |  | |  | |  | |  | |  | |  | |  | |  | |
| EXT Base | 0.156* | | 0.095 | | -0.298** | | 0.466** | |  | |  | |  | |  | |  | |  | |  | |  | |  | |  | |  | |  | |  | |  | |
| INT T1 | 0.413** | | 0.039 | | -0.126 | | 0.281** | | 0.197* | |  | |  | |  | |  | |  | |  | |  | |  | |  | |  | |  | |  | |  | |
| EXT T1 | 0.329** | | -0.162* | | -0.139* | | 0.146* | | 0.321** | | 0.39** | |  | |  | |  | |  | |  | |  | |  | |  | |  | |  | |  | |  | |
| INT T2 | 0.308** | | 0.104 | | 0.031 | | 0.261** | | 0.139 | | 0.615** | | 0.282** | |  | |  | |  | |  | |  | |  | |  | |  | |  | |  | |  | |
| EXT T2 | 0.322** | | -0.169* | | -0.092 | | 0.179* | | 0.303** | | 0.284** | | 0.659** | | 0.43** | |  | |  | |  | |  | |  | |  | |  | |  | |  | |  | |
| Phys | -0.103 | | -0.06 | | 0.121 | | -0.041 | | -0.052 | | -0.163* | | 0.019 | | -0.077 | | 0.001 | |  | |  | |  | |  | |  | |  | |  | |  | |  | |
| Nature | 0.047 | | -0.156* | | 0.053 | | -0.055 | | -0.05 | | -0.141* | | 0.059 | | -0.149 | | 0.002 | | 0.364** | |  | |  | |  | |  | |  | |  | |  | |  | |
| Outdoor | -0.03 | | -0.248** | | 0.044 | | -0.111 | | -0.018 | | -0.062 | | 0.081 | | -0.085 | | 0.101 | | 0.392** | | 0.161* | |  | |  | |  | |  | |  | |  | |  | |
| Screen | 0.049 | | 0.375** | | -0.062 | | 0.073 | | 0.143* | | 0.093 | | 0.18 | | 0.125 | | 0.032 | | -0.03 | | -0.024 | | -0.058 | |  | |  | |  | |  | |  | |  | |
| News | 0.156* | | 0.234** | | -0.028 | | -0.002 | | -0.039 | | 0.091* | | 0.092 | | -0.008 | | 0.024 | | 0.011 | | 0.083 | | 0.01 | | 0.55** | |  | |  | |  | |  | |  | |
| Sleep | -0.06 | | -0.338** | | 0.102 | | -0.153* | | -0.12 | | -0.023 | | -0.044 | | -0.02 | | -0.13 | | 0.081 | | 0.099 | | 0.075 | | -0.252** | | -0.15* | |  | |  | |  | |  | |
| Routine | -0.102 | | -0.037 | | 0.024 | | -0.009 | | 0.027 | | -0.072 | | -0.085 | | -0.006 | | -0.123 | | 0.104 | | 0.034 | | -0.046 | | -0.049 | | -0.136 | | 0.119 | |  | |  | |  | |
| Coping | 0.057 | | 0.074 | | 0.063 | | -0.105 | | -0.14* | | 0.023 | | 0.067 | | -0.031 | | 0.042 | | 0.247** | | 0.044 | | 0.182* | | 0.073 | | 0.203* | | 0.021 | | -0.109 | |  | |  | |
| Helping | -0.098 | | 0.07 | | 0.101 | | -0.015 | | -0.008 | | 0.017 | | -0.026 | | 0.081 | | 0.014 | | 0.051 | | -0.006 | | 0.1 | | 0.035 | | 0.05 | | 0.126 | | 0.001 | | 0.2* | |  | |

* denotes p < .05, ** denotes p < .01

**S4 Table: Associations between individual stressors and psychopathology at T1 and T2, correcting for continuous age, sex, income-to-needs ratio, and psychopathology prior to the pandemic.**

| Stressor | Internalizing T1 | | Internalizing T2 | | Externalizing T1 | | Externalizing T2 | |
| --- | --- | --- | --- | --- | --- | --- | --- | --- |
|  | β | *p* | β | *p* | β | *p* | β | *p* |
| Got sick with COVID-19 | **0.279** | **<.001** | **0.194** | **.008** | 0.097 | .124 | .085 | .238 |
| Had a parent or sibling get sick with COVID-19 | **0.160** | **.013** | -0.026 | .727 | 0.003 | .964 | -0.014 | .844 |
| Had another relative get sick with COVID-19 | -0.014 | .833 | -0.049 | .516 | 0.032 | .613 | 0.064 | .382 |
| Had a partner or close friend get sick with COVID-19 | 0.030 | .634 | -0.073 | .326 | 0.027 | .670 | -0.001 | .988 |
| Knew someone who died as a result of COVID-19 | 0.024 | .708 | -0.073 | .328 | **0.192** | **.002** | **0.180** | **.012** |
| Parent is a frontline worker (healthcare) | 0.048 | .456 | -0.038 | .611 | -0.007 | .918 | -0.065 | .373 |
| Parent is frontline worker (e.g. grocery) | 0.025 | .700 | 0.089 | . 229 | 0.067 | .288 | 0.039 | .593 |
| Felt lonely often | **0.357** | **<.001** | **0.297** | **<.001** | **0.263** | **<.001** | **0.220** | **.003** |
| Experienced discrimination related to the pandemic | 0.103 | .104 | *0.136* | *.068* | 0.034 | .593 | 0.068 | .346 |
| Difficult relationship with a parent that has gotten worse during the pandemic | **0.199** | **.002** | **0.184** | **.015** | **0.167** | **.009** | 0.098 | .179 |
| Difficult relationship with someone else in the home that has gotten worse | **0.160** | **.012** | 0.086 | .251 | **0.165** | **.009** | 0.037 | .603 |
| Experienced food insecurity during the pandemic | -0.019 | .783 | 0.073 | .381 | -0.087 | .206 | 0.113 | .161 |
| Parent lost a job during the pandemic | 0.075 | .258 | 0.014 | .862 | 0.043 | .502 | 0.066 | .372 |
| Significant financial losses due to the pandemic | 0.068 | .302 | 0.067 | .381 | 0.052 | .423 | **0.077** | **0.014** |
| Difficulty doing school work remotely | **0.274** | **<.001** | **0.158** | **.033** | **0.383** | **<.001** | **0.269** | **.001** |
| Noisy school work environment | -0.005 | .935 | 0.116 | .125 | 0.038 | .560 | -0.001 | .992 |
| Crowding in the home | 0.014 | .838 | 0.100 | .194 | -0.018 | .788 | *0.124* | *0.098* |

Significant associations are in bold and marginal associations are in italics

**S1 Fig. Age x Screen time interaction predicting internalizing symptoms.**

**
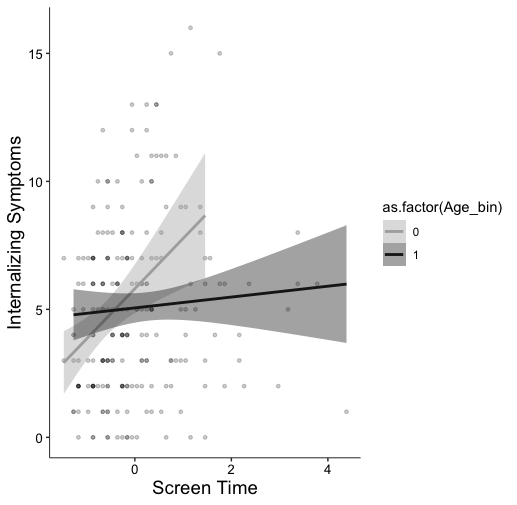
**

β= -1.953, *p* = .030

Children

Adolescents

A binary variable for children (7-10 years) and adolescents (13-15 years) was used for the simple slopes analysis and for visualization purposes. All analyses control for age, sex, and pre-pandemic psychopathology symptoms.

**The COVID-19 Experience Survey**

**Thank you for agreeing to participate in this research. This survey asks about your experiences related to the COVID-19 outbreak during the period of March 2020 and the resulting shelter-in-place orders that may have occurred in your community. Questions ask about how you and those close to you have been impacted in the areas of health, finances, and social and emotional functioning.**

**YOUR ANSWERS WILL BE KEPT STRICTLY CONFIDENTIAL.**

**The survey takes about 60 minutes. You may skip any questions you prefer not to answer. To thank you for your time, you will receive $50 via your choice of Venmo, Paypal or Gift Card when you are finished.**

*We are conducting this research to better understand how stressful experiences impact families. There are no direct benefits to you from participating in this research and there are few foreseeable risks associated with completing the survey. Your participation in this survey is completely voluntary. You do not need to complete the survey if you do not want to. Your choice whether or not to participate will not affect your current or future dealings with University of Washington or Harvard University. If you choose to complete the survey, you are free to stop the survey at any time.*

First, we’re going to ask you some questions about how things were ***before*** the coronavirus:

1. Before the outbreak, were you employed?
   1. Yes
   2. No
2. [If 1a]: What was your occupation?
   1. [Fill]
3. Before the outbreak, did you live with a partner?
   1. Yes
   2. No
4. [If 3a]: Was your partner employed?
   1. Yes
   2. No
5. [If 4a]: What was their occupation?
   1. [Fill]
6. Which of these categories best describes your total combined family income for the 12 months ***before*** the outbreak? This should include income (before taxes) from all sources, wages, rents from properties, social security, disability and/or veteran's benefits, unemployment benefits, workman's compensation, help from relatives (including child payments and alimony), and so on.
   1. $14,570 or less
   2. $14,571 – $18,310
   3. $18,311 – $22,050
   4. $22,051 – $25,790
   5. $25,791 – $29,530
   6. $29,531 – $33,270
   7. $33,271 – $37,010
   8. $37,011 – $39,200
   9. $39,201 – $48,200
   10. $48,201 – $58,400
   11. $58,401 – $75,000
   12. $75,001 – $100,000
   13. $100,001 – $150,000
   14. $150,000 or greater
   15. N/A or Prefer Not to Answer
7. Before the outbreak, were you ever hungry but did not eat because you could not afford to buy food?
   1. Never
   2. Rarely
   3. Sometimes
   4. Often
8. Did you ever eat less than you felt you should because you didn't have money to buy food?
   1. Never
   2. Rarely
   3. Sometimes
   4. Often
9. How often did you not have enough money to buy food?
   1. Never
   2. Rarely
   3. Sometimes
   4. Often
10. How often could you not afford to eat balanced meals?
    1. Never
    2. Rarely
    3. Sometimes
    4. Often
11. How often could you not afford to pay your rent or mortgage?
    1. Never
    2. Rarely
    3. Sometimes
    4. Often
12. How often could you not afford to pay for your utilities?
    1. Never
    2. Rarely
    3. Sometimes
    4. Often
13. Before the outbreak, how many individuals were living with you in your home?
    1. Number of adults: [Fill]
    2. Number of children: [Fill]
14. Before the outbreak, how many hours a day did you typically spend passively scrolling through social media?
    1. None
    2. 1 hour or less
    3. 1-2 hours
    4. 2-4 hours
    5. 4-6 hours
    6. 6 or more hours
15. How many hours a day did you typically spend passively browsing other non-news websites?
    1. None
    2. 1 hour or less
    3. 1-2 hours
    4. 2-4 hours
    5. 4-6 hours
    6. 6 or more hours
16. How many hours a day did you typically spend watching movies or shows for leisure?
    1. None
    2. 1 hour or less
    3. 1-2 hours
    4. 2-4 hours
    5. 4-6 hours
    6. 6 or more hours
17. How many hours a day did you typically spend reading books or magazines for leisure?
    1. None
    2. 1 hour or less
    3. 1-2 hours
    4. 2-4 hours
    5. 4-6 hours
    6. 6 or more hours
18. Before the coronavirus outbreak, how many hours a day did you typically spend actively socializing with people ***not*** in your household?
    1. None
    2. 1 hour or less
    3. 1-2 hours
    4. 2-4 hours
    5. 4-6 hours
    6. 6 or more hours
19. [If 18b-f]: Rank most commonly used methods of communication:
    1. Social media apps
    2. Texting
    3. Phone calls
    4. Video-chatting
    5. In-person
20. How many hours a day did you typically spend socializing with people ***who were*** in your household?
    1. None
    2. 1 hour or less
    3. 1-2 hours
    4. 2-4 hours
    5. 4-6 hours
    6. 6 or more hours
21. [If 20b-f]: Rank most commonly used methods of communication:
    1. Social media apps
    2. Texting
    3. Phone calls
    4. Video-chatting
    5. In-person
22. Before the coronavirus outbreak, were you a primary caretaker of individuals in your home?
    1. Yes
    2. No
23. [If 22a]: What is their relationship to you?
    1. Parent
    2. Child(ren)
    3. Partner
    4. Other [Fill]
24. Before the coronavirus outbreak, what did you typically do for physical exercise?
    1. Biking
    2. Running
    3. Dance
    4. Organized sport
    5. Swimming
    6. Yoga or pilates
    7. Aerobics or other cardio
    8. Other: [Fill in]
    9. I did not engage in physical exercise
25. [If selected 24a-h]: On average, how often did you exercise?
    1. Less than 1 day a week
    2. At least 1 day a week
    3. 2-3 days a week
    4. 4-5 days a week
    5. Every day or nearly every day
26. [If selected 24a-h]: On average, how long did you typically exercise for?
    1. Less than 30 minutes
    2. 30-60 minutes
    3. 60-90 minutes
    4. 90+ minutes
27. Before the coronavirus outbreak, how often did you spend time in outdoors green spaces (e.g., open spaces including parks, canals, nature areas, coastal or beach front, countryside, farmland)?
    1. Less than 1 day a week
    2. At least 1 day a week
    3. 2-3 days a week
    4. 4-5 days a week
    5. Every day or nearly every day
28. How often did you spend time outside your home for at least 30 minutes not including outdoor green spaces listed above (e.g., back yard, neighborhood street)?
    1. Less than 1 day a week
    2. At least 1 day a week
    3. 2-3 days a week
    4. 4-5 days a week
    5. Every day or nearly every day
29. On an average night, how well did you sleep before the coronavirus outbreak? My sleep quality was:
    1. Not good
    2. Somewhat good
    3. Mostly good
    4. Very good
30. On an average night, how many hours did you sleep?
    1. [fill] hours
31. If you experienced trouble sleeping, was it because of: (check all that apply or leave blank if no trouble sleeping):
    1. Trouble falling asleep
    2. Waking up during the night
    3. Waking up earlier than you wanted to
32. Before the coronavirus outbreak, did your days have a fairly consistent routine?
    1. Not at all, every day was different
    2. Somewhat, I did some things at the same time every day
    3. Mostly, I did most things at the same time every day
    4. Very much, I did everything at the same time every day
33. Did family members really help and support each other?
    1. Not at all
    2. Somewhat
    3. Mostly
    4. Very much
34. Was there was a feeling of togetherness in your family?
    1. Not at all
    2. Somewhat
    3. Mostly
    4. Very much
35. Did family members rarely criticize each other?
    1. Not at all
    2. Somewhat
    3. Mostly
    4. Very much

Now we’re going to ask you some questions about how things have been ***since*** the coronavirus pandemic started. **Specifically, we are interested in how things have been going in the past month**.

1. Do you believe you got sick with the coronavirus?
   1. No
   2. Possibly
   3. Yes
2. [If 36 b-c]: Did you get tested for the coronavirus?
   1. Did not want to get tested
   2. Wanted to get tested, but was unable
   3. Took a test and was positive
   4. Took a test and was negative
3. [If 36 b-c]: Did you experience symptoms?
   1. Yes
   2. No
4. [If 38 a]: For how long did you experience symptoms?
   1. 1-2 days
   2. 2-4 days
   3. 4-7 days
   4. 1-2 weeks
   5. More than 2 weeks
5. [If 36b-c]: Were you quarantined as a result?
   1. Yes
   2. No
6. [If 40 a]: For how long were you quarantined?
   1. 1-2 days
   2. 2-4 days
   3. 4-7 days
   4. 1-2 weeks
   5. More than 2 weeks
7. [If 36b-c]: Were you hospitalized? If so, for how long?
   1. Yes
   2. No
8. [If 42a]: For how long were you hospitalized?
   1. 1-2 days
   2. 2-4 days
   3. 4-7 days
   4. 1-2 weeks
   5. More than 2 weeks
9. Do you know anybody who has gotten sick with the coronavirus? If so, who? (Check all that apply)
   1. No one I know has gotten the coronavirus
   2. Child
   3. Parent
   4. Sibling
   5. Other relative (describe)
   6. Boyfriend/girlfriend/partner
   7. Close friend
   8. Coworker
   9. Acquaintance
   10. Other (describe)
10. [If 44b]: How serious was it for your child?
    1. Not serious (almost no symptoms)
    2. Mild symptoms (low fever, mild cough)
    3. Moderate symptoms (high fever, difficulty breathing)
    4. Severe symptoms (required hospitalization)
11. [If 44c]: How serious was it for your parent?
    1. Not serious (almost no symptoms)
    2. Mild symptoms (low fever, mild cough)
    3. Moderate symptoms (high fever, difficulty breathing)
    4. Severe symptoms (required hospitalization)
12. [If 44d]: How serious was it for your sibling? If more than one, describe the most serious.
    1. Not serious (almost no symptoms)
    2. Mild symptoms (low fever, mild cough)
    3. Moderate symptoms (high fever, difficulty breathing)
    4. Severe symptoms (required hospitalization)
13. [If 44e]: How serious was it for your other relative? If more than one, describe the most serious.
    1. Not serious (almost no symptoms)
    2. Mild symptoms (low fever, mild cough)
    3. Moderate symptoms (high fever, difficulty breathing)
    4. Severe symptoms (required hospitalization)
14. [If 44f]: How serious was it for your boyfriend/girlfriend/partner?
    1. Not serious (almost no symptoms)
    2. Mild symptoms (low fever, mild cough)
    3. Moderate symptoms (high fever, difficulty breathing)
    4. Severe symptoms (required hospitalization)
15. [If 44g]: How serious was it for your close friend? If more than one, describe the most serious.
    1. Not serious (almost no symptoms)
    2. Mild symptoms (low fever, mild cough)
    3. Moderate symptoms (high fever, difficulty breathing)
    4. Severe symptoms (required hospitalization)
16. [If 44h]: How serious was it for your coworker? If more than one, describe the most serious.
    1. Not serious (almost no symptoms)
    2. Mild symptoms (low fever, mild cough)
    3. Moderate symptoms (high fever, difficulty breathing)
    4. Severe symptoms (required hospitalization)
17. [If 44i]: How serious was it for your acquaintance? If more than one, describe the most serious.
    1. Not serious (almost no symptoms)
    2. Mild symptoms (low fever, mild cough)
    3. Moderate symptoms (high fever, difficulty breathing)
    4. Severe symptoms (required hospitalization)
18. [If 44j]: How serious was it for your [other fill in]? If more than one, describe the most serious.
    1. Not serious (almost no symptoms)
    2. Mild symptoms (low fever, mild cough)
    3. Moderate symptoms (high fever, difficulty breathing)
    4. Severe symptoms (required hospitalization)
19. [If 44b-j]: Do you know anybody who has died as a result of the coronavirus? If so, what is this person’s relationship to you? (Check all that apply)
    1. No
    2. Child
    3. Parent
    4. Sibling
    5. Other relative (describe)
    6. Boyfriend/girlfriend/partner
    7. Close friend
    8. Coworker
    9. Acquaintance
    10. Other (describe)
20. Has your employment status changed?
    1. Yes
    2. No
21. [If 55 a]: How has your employment status changed?
    1. Gained employment
    2. Laid off
    3. Reduced hours
    4. Remote work
    5. Went out of business
    6. Other: [FILL IN]
22. [If 55 a] How long have you been experiencing any of the above changes in employment? [For each 56a-f selected above]:
    1. Less than 1 week
    2. 1-2 weeks
    3. 2-3 weeks
    4. 3-4 weeks
    5. 1-2 months
    6. 2-3 months
    7. 3 or more months
23. Are you able to work from home?
    1. Yes
    2. No
24. Are you currently living with a partner?
    1. Yes
    2. No
25. Did their employment status change?
    1. Yes
    2. No
26. [If 60a]: How has their employment status changed?
    1. Gained employment
    2. Laid off
    3. Reduced hours
    4. Remote work
    5. Went out of business
    6. Other: [FILL IN]
27. [If 60a] How long have you been experiencing any of the above changes in employment? [For each 61a-f selected above]:
    1. Less than 1 week
    2. 1-2 weeks
    3. 2-3 weeks
    4. 3-4 weeks
    5. 1-2 months
    6. 2-3 months
    7. 3 or more months
28. Is your partner able to work from home?
    1. Yes
    2. No
29. Did you and/or your partner experience significant financial losses as a result of the outbreak and surrounding events?
    1. Yes
    2. No
30. [If 64 a]: How extensive were these losses?
    1. Minimal (will not require any adjustment in lifestyle or future planning and goals)
    2. Moderate (will require major lifestyle changes and some minor adjustments in future planning and goals)
    3. Severe (will require major lifestyle changes, future planning and goals are at risk)
    4. Devastating (complete loss of financial assets, will require continued or soliciting dependence on others or government)
31. [If 64 a]: What were these financial losses due to (check all that apply)?
    1. Stock market losses
    2. Loss of business
    3. Work hours reduced
    4. Loss of employment
    5. Other: [fill in]
32. Have you had to terminate an employee either at work or in your home (e.g., nanny, housekeeper) as a result of financial losses due to the coronavirus?
    1. Yes
    2. No
33. In the last month, have you been evicted or otherwise forced to leave your home because of financial reasons?
    1. Yes
    2. No
34. Please provide the following information about your *current* home:
    1. Number of bedrooms [FILL IN]
    2. Number of bathrooms [FILL IN]
    3. Square-footage (approximate) [FILL IN]
35. In the last month, how many individuals were living with you in your home?
    1. Number of adults: [Fill]
    2. Number of children: [Fill]
36. In the past month, have you been a primary caretaker of individuals in your home?
    1. Yes
    2. No
37. [If 71a]: What is their relationship to you?
    1. Parent
    2. Child(ren)
    3. Partner
    4. Other [Fill]
38. Do you have a serious physical or mental illness?
    1. Physical
    2. Mental
    3. No
39. [If 74a-b]: Have there been disruptions in needed medical care due to the outbreak?
    1. Yes
    2. No
40. Does someone else in the household have a serious physical or mental illness?
    1. Physical
    2. Mental
    3. No
41. [If 75a-b]: Have there been disruptions in needed medical care for that person due to the outbreak?
    1. Yes
    2. No
42. Do you have reliable internet access?
    1. Not at all
    2. A little
    3. Somewhat
    4. Very
43. Which of these categories best describes your anticipated total combined family income for the 12 months ***after the outbreak***? This should include income (before taxes) from all sources, wages, rents from properties, social security, disability and/or veteran's benefits, unemployment benefits, workman's compensation, help from relatives (including child payments and alimony), and so on.
    1. $14,570 or less
    2. $14,571 – $18,310
    3. $18,311 – $22,050
    4. $22,051 – $25,790
    5. $25,791 – $29,530
    6. $29,531 – $33,270
    7. $33,271 – $37,010
    8. $37,011 – $39,200
    9. $39,201 – $48,200
    10. $48,201 – $58,400
    11. $58,401 – $75,000
    12. $75,001 – $100,000
    13. $100,001 – $150,000
    14. $150,000 or greater
    15. N/A or Prefer Not to Answer
44. In the last month, were you ever hungry but did not eat because you could not afford to buy food?
    1. Never
    2. Rarely
    3. Sometimes
    4. Often
45. Did you ever eat less than you felt you should because you didn't have money to buy food?
    1. Never
    2. Rarely
    3. Sometimes
    4. Often
46. How often did you not have enough money to buy food?
    1. Never
    2. Rarely
    3. Sometimes
    4. Often
47. How often could you not afford to eat balanced meals?
    1. Never
    2. Rarely
    3. Sometimes
    4. Often
48. How often could you not afford to pay your rent or mortgage?
    1. Never
    2. Rarely
    3. Sometimes
    4. Often
49. How often could you not afford to pay for your utilities?
    1. Never
    2. Rarely
    3. Sometimes
    4. Often
50. Are you or your partner a healthcare provider?
    1. Yes
    2. No, neither myself nor my partner are healthcare providers.
51. [If 85 a]: Have you or your partner continued to go to work since the beginning of the outbreak?
    1. Work has continued and I have continued to go to work.
    2. Work has continued and I have ***not*** continued to go to work.
    3. Work has not continued.
52. Do you or your partner work in a grocery store, pharmacy, or other retail establishment?
    1. Yes
    2. No
53. [If 87 a]: Have you or your partner continued to go to work since the beginning of the outbreak?
    1. Work has continued and I have continued to go to work.
    2. Work has continued and I have ***not*** continued to go to work.
    3. Work has not continued.
54. Is anyone in your household immunocompromised (e.g., weakened immune system due to other medical conditions)?
    1. Yes
    2. No
55. [If 89 a]: What is your relationship to that person?
    1. Me
    2. My partner
    3. My child(ren)
    4. My parent(s)
    5. Other: [fill in].
56. Have you been social distancing (e.g., putting at least 6 feet of physical distance between yourself and other people who do not live in your household)?
    1. Yes
    2. No
57. [If 91a]: For how long have you been social distancing?
    1. 1-2 days
    2. 2-4 days
    3. 4-7 days
    4. 1-2 weeks
    5. More than 2 weeks
58. On average, how much have you and your family followed social distancing recommendations?
    1. We have made no changes to our behavior.
    2. We have made minor changes to reduce social contact (e.g., going out less, seeing fewer friends)
    3. We have made major changes to reduce physical social contact (e.g., not going to school / work, limiting contact with people outside our home)
    4. Completely changed (e.g., staying inside almost all the time, only going out for necessities, and keeping my physical distance from other people when we do)
59. On average, how much have members of your community followed social distancing recommendations?
    1. They have made no changes to their behavior.
    2. They have made minor changes to reduce social contact (e.g., going out less, seeing fewer friends)
    3. They have made major changes to reduce physical social contact (e.g., not going to school / work, limiting contact with people outside their home)
    4. Completely changed (e.g., staying inside almost all the time, only going out for necessities, and keeping their physical distance from other people when they do)
60. Is your child staying home from school?
    1. Yes: school closed
    2. Yes: school remained open, but I’m keeping my child home
    3. No
61. When did your children begin staying home from school?
    1. [DATE]
62. Do you assist your child on academic activities? May vary based on age-appropriateness.
    1. Yes
    2. No
63. [If 97a]: How many hours a day do you typically spend on academic activities with your child?
    1. Hours [FILL]
64. [If 97a] Where did you obtain sources for academic instruction or guidance?
    1. I made the materials myself
    2. Child’s school
    3. Family or friends
    4. Organization: [Fill]
65. Does your child need to use the internet to do their school work?
    1. Yes
    2. No
66. Does your child have their own computer or tablet to do their schoolwork on? If so, how did they get this device?
    1. Yes, they had it before the outbreak
    2. Yes, we bought it after the outbreak
    3. Yes, the school provided it after the outbreak
    4. Yes, someone donated it after the outbreak
    5. No
67. In the last month, approximately how much of the day do you typically spend watching or reading the news coverage about the outbreak through formal news sources (e.g., news channel on TV, news source webpage)?
    1. None
    2. 1 hour or less
    3. 1-2 hours
    4. 2-4 hours
    5. 4-6 hours
    6. 6 or more hours
68. In the last month, approximately how much of the day do you typically spend watching or reading the news coverage about the outbreak through informal news sources (e.g., social media posts, blogs, talk shows; does not include links from social media to actual news source articles)?
    1. None
    2. 1 hour or less
    3. 1-2 hours
    4. 2-4 hours
    5. 4-6 hours
    6. 6 or more hours
69. In the last month, how many hours a day do you typically spend passively scrolling through social media?
    1. None
    2. 1 hour or less
    3. 1-2 hours
    4. 2-4 hours
    5. 4-6 hours
    6. 6 or more hours
70. How many hours a day do you typically spend passively browsing other non-news websites?
    1. None
    2. 1 hour or less
    3. 1-2 hours
    4. 2-4 hours
    5. 4-6 hours
    6. 6 or more hours
71. How many hours a day do you typically spend watching movies or shows for leisure?
    1. None
    2. 1 hour or less
    3. 1-2 hours
    4. 2-4 hours
    5. 4-6 hours
    6. 6 or more hours
72. How many hours a day do you typically spend reading books or magazines for leisure?
    1. None
    2. 1 hour or less
    3. 1-2 hours
    4. 2-4 hours
    5. 4-6 hours
    6. 6 or more hours
73. In the last month, how many hours a day have you been spending actively socializing either in person or digitally with people ***not*** in your household?
    1. None
    2. 1 hour or less
    3. 1-2 hours
    4. 2-4 hours
    5. 4-6 hours
    6. 6 or more hours
74. [If 104b-f:] Rank most commonly used methods of communication:
    1. Social media apps
    2. Texting
    3. Phone calls
    4. Video-chatting
    5. In-person
75. In the last month, how many hours a day have you been spending actively socializing either in person or digitally with people ***who are*** in your household?
    1. None
    2. 1 hour or less
    3. 1-2 hours
    4. 2-4 hours
    5. 4-6 hours
    6. 6 or more hours
76. [If 106b-f]: Rank most commonly used methods of communication:
    1. Social media apps
    2. Texting
    3. Phone calls
    4. Video-chatting
    5. In-person
77. In the last month, do you feel more or less connected to the following people:
    1. Close Friends
       1. Much less connected
       2. A little less connected
       3. The same
       4. A little more connected
       5. Much more connected
    2. Other friends
       1. Much less connected
       2. A little less connected
       3. The same
       4. A little more connected
       5. Much more connected
    3. Family in household
       1. Much less connected
       2. A little less connected
       3. The same
       4. A little more connected
       5. Much more connected
    4. Family not in household
       1. Much less connected
       2. A little less connected
       3. The same
       4. A little more connected
       5. Much more connected
    5. Your community
       1. Much less connected
       2. A little less connected
       3. The same
       4. A little more connected
       5. Much more connected
78. In the last month, how much have you missed being with people ***who do not live with you:***
    1. Close Friends
       1. Not at all
       2. A little
       3. Somewhat
       4. Very
    2. Other friends
       1. Not at all
       2. A little
       3. Somewhat
       4. Very
    3. Family
       1. Not at all
       2. A little
       3. Somewhat
       4. Very
    4. Coworkers
       1. Not at all
       2. A little
       3. Somewhat
       4. Very
    5. Community
       1. Not at all
       2. A little
       3. Somewhat
       4. Very
79. In the last month, how often have you felt lonely?
    1. Never
    2. Once
    3. Several Times
    4. A few times a week
    5. Nearly every day
80. In the last month, what have you typically been doing for physical exercise?
    1. Biking
    2. Running
    3. Dance
    4. Organized sport
    5. Swimming
    6. Yoga or pilates
    7. Aerobics or other cardio
    8. Other: [Fill in]
    9. I have not engaged in physical exercise
81. [If selected 115a-h]: On average, how often have you exercised?
    1. Less than 1 day a week
    2. At least 1 day a week
    3. 2-3 days a week
    4. 4-5 days a week
    5. Every day or nearly every day
82. [If selected 115a-h]: On average, how long have you typically exercised for?
    1. Less than 30 minutes
    2. 30-60 minutes
    3. 60-90 minutes
    4. 90+ minutes
83. In the last month, how often have you spent time in outdoors green spaces (e.g., open spaces including parks, canals, nature areas, coastal or beach front, countryside, farmland)?
    1. Less than 1 day a week
    2. At least 1 day a week
    3. 2-3 days a week
    4. 4-5 days a week
    5. Every day or nearly every day
84. How often have you spent time outside your home for at least 30 minutes not including outdoor green spaces listed above (e.g., back yard, neighborhood street)?
    1. Less than 1 day a week
    2. At least 1 day a week
    3. 2-3 days a week
    4. 4-5 days a week
    5. Every day or nearly every day
85. In the past month, how well have you been sleeping? My sleep quality was:
    1. Not good
    2. Somewhat good
    3. Mostly good
    4. Very good
86. On an average night, how many hours do you sleep?
    1. [fill] hours
87. If you experienced trouble sleeping, was it because of (check all that apply or leave blank if no trouble sleeping):
    1. Trouble falling asleep
    2. Waking up during the night
    3. Waking up earlier than you wanted to
88. In the last month, have your days had a fairly consistent routine?
    1. Not at all, every day was different
    2. Somewhat, I did some things at the same time every day
    3. Mostly, I did most things at the same time every day
    4. Very much, I did everything at the same time every day
89. Have family members really helped and supported each other?
    1. Not at all
    2. Somewhat
    3. Mostly
    4. Very much
90. Has there been a feeling of togetherness in your family?
    1. Not at all
    2. Somewhat
    3. Mostly
    4. Very much
91. Have family members rarely criticized each other?
    1. Not at all
    2. Somewhat
    3. Mostly
    4. Very much
92. Have you had a discussion about the coronavirus with your child?
    1. Yes
    2. No
93. [If 127a]: How difficult did you find this conversation?
    1. Not at all difficult
    2. A little difficult
    3. Somewhat difficult
    4. Very difficult
94. Do you have a difficult relationship with someone who lives in your home? If so, who? Check all that apply.
    1. Yes- Partner
    2. Yes- Child
    3. Yes- Other [fill in]
    4. No
95. [If 129a-c]: Has being at home with this person more changed things in your relationship? [logic, appears for each relationship selected]
    1. Yes gotten worse
    2. Yes gotten better
    3. No stayed about the same
96. [If 129a-c]: How upsetting is it to be at home more often with this person?
    1. Not at all upsetting
    2. A little upsetting
    3. Somewhat upsetting
    4. Very upsetting
97. Have you or anyone in your household experienced any racism, prejudice, or discrimination (i.e., being treated unfairly because of some aspect of your identity) related to the coronavirus outbreak?
    1. No
    2. Yes
       1. [if b selected] Racial slur
       2. [if b selected] Avoided because of my/their race or ethnicity
       3. [if b selected] Attacked because of my/their race or ethnicity
       4. [if b selected] Explain [Fill in]
98. How have your community leaders been discussing the coronavirus outbreak (government, religious, or otherwise)?
    1. Not at all seriously, they say it’s not a big deal
    2. A little seriously, they say we just have to be a little more careful
    3. Somewhat seriously, they say we we should change a lot of our behavior
    4. Very seriously, they say that it’s very dangerous and it’s important to change our behavior
99. Do you trust the government or leaders of your community to take the appropriate steps to manage your safety?
    1. Not at all
    2. A little
    3. Somewhat
    4. Very
100. How well do you feel like you understand the current pandemic and the reasoning behind social distancing?
     1. Not at all
     2. A little
     3. Somewhat
     4. Very
101. For each item below, indicate ***the most worried you have felt in the last month*** using the sliding scale from ‘not at all worried’ to ‘extremely worried.’

|  | **1** | **2** | **3** | **4** | **5** | **6** | **7** | **8** | **9** | **10** |
| --- | --- | --- | --- | --- | --- | --- | --- | --- | --- | --- |
| You would become seriously ill or die |  |  |  |  |  |  |  |  |  |  |
| One of your children would become seriously ill or die |  |  |  |  |  |  |  |  |  |  |
| Someone else you care about would be become seriously ill or die |  |  |  |  |  |  |  |  |  |  |
| You would be unable to access care if you were sick |  |  |  |  |  |  |  |  |  |  |
| Someone you care about would be unable to access care if they got sick |  |  |  |  |  |  |  |  |  |  |
| Infected items would be brought into your home |  |  |  |  |  |  |  |  |  |  |
| Need to disinfect items brought into your home |  |  |  |  |  |  |  |  |  |  |
| Your family wouldn’t be able to pay bills |  |  |  |  |  |  |  |  |  |  |
| Your family wouldn’t have enough money for basic needs like food and shelter |  |  |  |  |  |  |  |  |  |  |
| Your family would lose their housing |  |  |  |  |  |  |  |  |  |  |
| You would lose your job |  |  |  |  |  |  |  |  |  |  |
| Your partner would lose their job |  |  |  |  |  |  |  |  |  |  |
| Your family wouldn’t be able to get enough food |  |  |  |  |  |  |  |  |  |  |
| Your family wouldn’t be able to get other necessities like toilet paper and cleaning supplies |  |  |  |  |  |  |  |  |  |  |
| Your family wouldn’t be able to get medicine |  |  |  |  |  |  |  |  |  |  |
| Someone else that you care about wouldn’t be able to pay bills |  |  |  |  |  |  |  |  |  |  |
| Someone else that you care about wouldn’t have enough money for basic needs like food and shelter |  |  |  |  |  |  |  |  |  |  |
| Someone else that you care about would lose their housing |  |  |  |  |  |  |  |  |  |  |
| Someone else that you care about would lose their job(s) |  |  |  |  |  |  |  |  |  |  |
| Someone else that you care about wouldn’t be able to get enough food |  |  |  |  |  |  |  |  |  |  |
| Someone else that you care about wouldn’t be able to get other necessities like toilet paper and cleaning supplies |  |  |  |  |  |  |  |  |  |  |
| Someone else that you care about wouldn’t be able to get medicine |  |  |  |  |  |  |  |  |  |  |
| Something bad would happen if you went outside |  |  |  |  |  |  |  |  |  |  |
| That you wouldn’t be able to perform well at work |  |  |  |  |  |  |  |  |  |  |
| That you made someone else sick with the coronavirus |  |  |  |  |  |  |  |  |  |  |
| That you or someone you know would be the victim of racism or discrimination |  |  |  |  |  |  |  |  |  |  |
|  |  |  |  |  |  |  |  |  |  |  |
|  |  |  |  |  |  |  |  |  |  |  |

1. In the last month, how have you dealt with any stress or anxiety related to the coronavirus (check all that apply)?
   1. Watched/read news
   2. Tried to distract yourself
   3. Thought about all the details of the problem
   4. Talked to family or friends
   5. Exercised
   6. Meditated
   7. Sought counseling from a therapist or religious leader
   8. Self-care activities
      1. Explain [Fill in]
   9. Other
      1. Explain [Fill in]
2. Have you done any of the following since the outbreak?
   1. Volunteered time at hospitals
   2. Donated/prepared food
   3. Donated money/supplies
   4. Gave shelter to displaced people
   5. Prayed/prayer group/prayer vigil
   6. Wrote letters or contacted isolated older people?
   7. Cheered on health care workers
   8. Other way of helping (specify _____________________)
3. Taking everything into consideration, what was the most stressful part of the coronavirus outbreak and subsequent social distancing/quarantine for you personally?
   1. [Fill in]
4. Is there anything we should know about the psychological effects of the outbreak that was not covered in this survey? If no, leave blank.
   1. [Fill in]

**The COVID-19 Experience Survey**

**Thank you for agreeing to participate in this research. This survey asks about your experiences related to the COVID-19 outbreak during the period of March 2020 and the resulting shelter-in-place orders that may have occurred in your community. Questions ask about how you and those close to you have been impacted in the areas of health and finances, as well as social, and emotional functioning.**

**YOUR ANSWERS WILL BE KEPT STRICTLY CONFIDENTIAL.**

**The survey takes about 60 minutes. You may skip any questions you prefer not to answer. To thank you for your time, you will receive $50 via your choice of Venmo, Paypal or Gift Card when you are finished.**

*We are conducting this research to better understand how stressful experiences impact families. There are no direct benefits to you from participating in this research and there are few foreseeable risks associated with completing the survey. Your participation in this survey is completely voluntary. You do not need to complete the survey if you do not want to. Your choice whether or not to participate will not affect your current or future dealings with University of Washington or Harvard University. If you choose to complete the survey, you are free to stop the survey at any time.*

First, we’re going to ask you some questions about how things were ***before*** the coronavirus:

1. What types of regular, organized group activities did you participate in during or outside of school? (Check all that apply)
   1. School clubs
   2. Sports / athletic activity (e.g., soccer, dance, running)
   3. Musical or artistic activities
   4. Other: [Fill]
   5. I did not participate in any
2. [If 1a]: How many days a week did you participate in school clubs?
   1. Less than 1 day a week
   2. At least 1 day a week
   3. 2-3 days a week
   4. 4-5 days a week
   5. Every day or nearly every day
3. [If 1b]: How many days a week did you participate in sports / athletic activity?
   1. Less than 1 day a week
   2. At least 1 day a week
   3. 2-3 days a week
   4. 4-5 days a week
   5. Every day or nearly every day
4. [If 1c]: How many days a week did you participate in musical or artistic activities?
   1. Less than 1 day a week
   2. At least 1 day a week
   3. 2-3 days a week
   4. 4-5 days a week
   5. Every day or nearly every day
5. [If 1d]: How many days a week did you participate in [other filled]?
   1. Less than 1 day a week
   2. At least 1 day a week
   3. 2-3 days a week
   4. 4-5 days a week
   5. Every day or nearly every day
6. Before the coronavirus outbreak, how often did you typically see your friends (aside from in class)?
   1. Never
   2. Less than once a week
   3. Once a week
   4. 2-3 times a week
   5. Once a day
   6. Multiple times a day
7. How often did you typically talk to friends on the phone?
   1. Never
   2. Less than once a week
   3. Once a week
   4. 2-3 times a week
   5. Once a day
   6. Multiple times a day
8. How often did you typically text with friends?
   1. Never
   2. Less than once a week
   3. Once a week
   4. 2-3 times a week
   5. Once a day
   6. Multiple times a day
9. How often did you typically talk to your friends on other messaging apps (WhatsApp, etc.)?
   1. Never
   2. Less than once a week
   3. Once a week
   4. 2-3 times a week
   5. Once a day
   6. Multiple times a day
10. How often did you typically talk to your friends on social media (SnapChat, Instagram, Facebook, etc.)?
    1. Never
    2. Less than once a week
    3. Once a week
    4. 2-3 times a week
    5. Once a day
    6. Multiple times a day
11. Before the coronavirus outbreak, how often did you typically see your parents?
    1. Never
    2. Less than once a week
    3. Once a week
    4. 2-3 times a week
    5. Once a day
    6. Multiple times a day
12. How often did you typically talk to your parents on the phone?
    1. Never
    2. Less than once a week
    3. Once a week
    4. 2-3 times a week
    5. Once a day
    6. Multiple times a day
13. How often did you typically text with your parents?
    1. Never
    2. Less than once a week
    3. Once a week
    4. 2-3 times a week
    5. Once a day
    6. Multiple times a day
14. How often did you typically talk to your parents on other messaging apps (WhatsApp, etc.)?
    1. Never
    2. Less than once a week
    3. Once a week
    4. 2-3 times a week
    5. Once a day
    6. Multiple times a day
15. How often did you typically talk to your parents on social media (SnapChat, Instagram, Facebook, etc.)?
    1. Never
    2. Less than once a week
    3. Once a week
    4. 2-3 times a week
    5. Once a day
    6. Multiple times a day
16. Before the coronavirus outbreak, how often did your peers leave you out of an ***online*** group activity or conversation that you really wanted to be included in (group chat, group photo, etc.)?
    1. Never
    2. Less than once a week
    3. Once a week
    4. 2-3 times a week
    5. Once a day
    6. Multiple times a day
17. How often did a peer threaten to hurt you or beat you up using an ***online*** medium (texting, social media app, photo caption, etc.)?
    1. Never
    2. Less than once a week
    3. Once a week
    4. 2-3 times a week
    5. Once a day
    6. Multiple times a day
18. How often did a peer tease you in a mean way saying rude things or calling you bad names using an ***online*** medium (texting, social media app, photo caption, etc.)?
    1. Never
    2. Less than once a week
    3. Once a week
    4. 2-3 times a week
    5. Once a day
    6. Multiple times a day
19. How often did a peer try to damage your social reputation using an ***online*** medium (texting, social media app, photo caption, etc.)?
    1. Never
    2. Less than once a week
    3. Once a week
    4. 2-3 times a week
    5. Once a day
    6. Multiple times a day
20. How often did a peer stop responding to you on an ***online*** medium (texting, social media app, etc.)?
    1. Never
    2. Less than once a week
    3. Once a week
    4. 2-3 times a week
    5. Once a day
    6. Multiple times a day
21. How often did a peer block you using an ***online*** medium (texting, social media app, etc.)?
    1. Never
    2. Less than once a week
    3. Once a week
    4. 2-3 times a week
    5. Once a day
    6. Multiple times a day
22. Before the coronavirus outbreak, how many hours a day did you typically spend passively scrolling through social media?
    1. None
    2. 1 hour or less
    3. 1-2 hours
    4. 2-4 hours
    5. 4-6 hours
    6. 6 or more hours
23. How many hours a day did you typically spend passively browsing other non-news websites?
    1. None
    2. 1 hour or less
    3. 1-2 hours
    4. 2-4 hours
    5. 4-6 hours
    6. 6 or more hours
24. How many hours a day did you typically spend watching movies or shows for leisure?
    1. None
    2. 1 hour or less
    3. 1-2 hours
    4. 2-4 hours
    5. 4-6 hours
    6. 6 or more hours
25. How many hours a day did you typically spend reading books or magazines for leisure?
    1. None
    2. 1 hour or less
    3. 1-2 hours
    4. 2-4 hours
    5. 4-6 hours
    6. 6 or more hours
26. Before the coronavirus outbreak, did you have an adult in your life you could turn to for emotional support?
    1. Yes
    2. No
27. [If 26a]: What’s their relationship to you? (Check all that apply)
    1. Parent
    2. Other family member
    3. Family friend
    4. Neighbor
    5. Coach
    6. Teacher
    7. Other mentor: [Fill]
28. [If 26a]: Think about the person you’d turn to the most for emotional support, how often did you talk to them?
    1. Every few months
    2. Every few weeks
    3. At least once a week
    4. Multiple times a week
29. Before the coronavirus outbreak, how many hours a day did you typically spend actively socializing either in person or digitally with people ***not*** in your household?
    1. None
    2. 1 hour or less
    3. 1-2 hours
    4. 2-4 hours
    5. 4-6 hours
    6. 6 or more hours
30. [If 29b-f]: Rank most commonly used methods of communication:
    1. Social media apps
    2. Texting
    3. Phone calls
    4. Video-chatting
    5. In-person
31. How many hours a day did you typically spend actively socializing either in person or digitally with people ***who are*** in your household?
    1. None
    2. 1 hour or less
    3. 1-2 hours
    4. 2-4 hours
    5. 4-6 hours
    6. 6 or more hours
32. [If 31b-f]: Rank most commonly used methods of communication:
    1. Social media apps
    2. Texting
    3. Phone calls
    4. Video-chatting
    5. In-person
33. Before the coronavirus, what did you typically do for physical exercise?
    1. Biking
    2. Running
    3. Dance
    4. Organized sport
    5. Swimming
    6. Yoga or pilates
    7. Aerobics or other cardio
    8. Other: [Fill in]
    9. I did not engage in physical exercise
34. [If selected 33a-h]: On average, how often did you exercise?
    1. Less than 1 day a week
    2. At least 1 day a week
    3. 2-3 days a week
    4. 4-5 days a week
    5. Every day or nearly every day
35. [If selected 33a-h]: On average, how long did you typically exercise for?
    1. Less than 30 minutes
    2. 30-60 minutes
    3. 60-90 minutes
    4. 90+ minutes
36. Before the coronavirus outbreak, how often did you spend time in outdoors green spaces (e.g., open spaces including parks, canals, nature areas, coastal or beach front, countryside, farmland)?
    1. Less than 1 day a week
    2. At least 1 day a week
    3. 2-3 days a week
    4. 4-5 days a week
    5. Every day or nearly every day
37. How often did you spend time outside your home for at least 30 minutes not including outdoor green spaces listed above (e.g., back yard, neighborhood street)?
    1. Less than 1 day a week
    2. At least 1 day a week
    3. 2-3 days a week
    4. 4-5 days a week
    5. Every day or nearly every day
38. On an average night, how well did you sleep before the coronavirus outbreak? My sleep quality was:
    1. Not good
    2. Somewhat good
    3. Mostly good
    4. Very good
39. On an average night, how many hours did you sleep?
    1. [fill] hours
40. If you experienced trouble sleeping, was it because of (check all that apply):
    1. Trouble falling asleep
    2. Waking up during the night
    3. Waking up earlier than you wanted to
    4. I have no trouble sleeping
41. Before the coronavirus outbreak, did your days have a fairly consistent routine?
    1. No at all, every day was different
    2. Somewhat, I did some things at the same time every day
    3. Mostly, I did most things at the same time every day
    4. Very much, I did everything at the same time every day
42. Did family members really help and support each other?
    1. Not at all
    2. Somewhat
    3. Mostly
    4. Very much
43. Was there was a feeling of togetherness in your family?
    1. Not at all
    2. Somewhat
    3. Mostly
    4. Very much
44. Did family members rarely criticize each other?
    1. Not at all
    2. Somewhat
    3. Mostly
    4. Very much

Now we’re going to ask you some questions about how things have been ***since*** the coronavirus pandemic started. **Specifically, we are interested in how things have been going over the past month**.

1. Do you believe you got sick with the coronavirus?
   1. No
   2. Possibly
   3. Yes
2. [If 45 b-c]: Did you get tested for the coronavirus?
   - 1. Did not want to get tested
     2. Wanted to get tested, but was unable
     3. Took a test and was positive
     4. Took a test and was negative
3. [If 45 b-c]: Did you experience symptoms?
   1. Yes
   2. No
4. [If 47 a]: For how long did you experience symptoms?
   1. 1-2 days
   2. 2-4 days
   3. 4-7 days
   4. 1-2 weeks
   5. More than 2 weeks
5. [If 45b-c]: Were you quarantined as a result?
   1. Yes
   2. No
6. [If 49 a]: For how long were you quarantined?
   1. 1-2 days
   2. 2-4 days
   3. 4-7 days
   4. 1-2 weeks
   5. More than 2 weeks
7. [If 45b-c]: Were you hospitalized?
   1. Yes
   2. No
8. [If 51a]: For how long were you hospitalized?
   1. 1-2 days
   2. 2-4 days
   3. 4-7 days
   4. 1-2 weeks
   5. More than 2 weeks
9. Do you know anybody who has gotten sick with the coronavirus? If so, who? (Check all that apply)
   1. No one I know has gotten the coronavirus
   2. Parent
   3. Sibling
   4. Other relative (describe)
   5. Boyfriend/girlfriend/partner
   6. Close friend
   7. Classmate
   8. Acquaintance
   9. Teacher
   10. Other (describe)
10. [If 53b]: How serious was it for your parent?
    1. Not serious (almost no symptoms)
    2. Mild symptoms (low fever, mild cough)
    3. Moderate symptoms (high fever, difficulty breathing)
    4. Severe symptoms (required hospitalization)
11. [If 53c]: How serious was it for your sibling? If more than one, describe the most serious.
    1. Not serious (almost no symptoms)
    2. Mild symptoms (low fever, mild cough)
    3. Moderate symptoms (high fever, difficulty breathing)
    4. Severe symptoms (required hospitalization)
12. [If 53d]: How serious was it for your other relative? If more than one, describe the most serious.
    1. Not serious (almost no symptoms)
    2. Mild symptoms (low fever, mild cough)
    3. Moderate symptoms (high fever, difficulty breathing)
    4. Severe symptoms (required hospitalization)
13. [If 53e]: How serious was it for your boyfriend/girlfriend/partner?
    1. Not serious (almost no symptoms)
    2. Mild symptoms (low fever, mild cough)
    3. Moderate symptoms (high fever, difficulty breathing)
    4. Severe symptoms (required hospitalization)
14. [If 53f]: How serious was it for your close friend? If more than one, describe the most serious.
    1. Not serious (almost no symptoms)
    2. Mild symptoms (low fever, mild cough)
    3. Moderate symptoms (high fever, difficulty breathing)
    4. Severe symptoms (required hospitalization)
15. [If 53g]: How serious was it for your classmate? If more than one, describe the most serious.
    1. Not serious (almost no symptoms)
    2. Mild symptoms (low fever, mild cough)
    3. Moderate symptoms (high fever, difficulty breathing)
    4. Severe symptoms (required hospitalization)
16. [If 53h]: How serious was it for your acquaintance? If more than one, describe the most serious.
    1. Not serious (almost no symptoms)
    2. Mild symptoms (low fever, mild cough)
    3. Moderate symptoms (high fever, difficulty breathing)
    4. Severe symptoms (required hospitalization)
17. [If 53i]: How serious was it for your teacher? If more than one, describe the most serious.
    1. Not serious (almost no symptoms)
    2. Mild symptoms (low fever, mild cough)
    3. Moderate symptoms (high fever, difficulty breathing)
    4. Severe symptoms (required hospitalization)
18. [If 53j]: How serious was it for your [other fill in]? If more than one, describe the most serious.
    1. Not serious (almost no symptoms)
    2. Mild symptoms (low fever, mild cough)
    3. Moderate symptoms (high fever, difficulty breathing)
    4. Severe symptoms (required hospitalization)
19. [If 53b-j]: Do you know anybody who has died as a result of the coronavirus? If so, what is this person’s relationship to you? (Check all that apply)
    1. No
    2. Parent
    3. Sibling
    4. Other relative (describe)
    5. Boyfriend/girlfriend/partner
    6. Close friend
    7. Classmate
    8. Acquaintance
    9. Teacher
    10. Other (describe)
20. In the last month, approximately how much of the day do you typically spend watching or reading the news coverage about the outbreak through formal news sources (e.g., news channel on TV, news source webpage)?
    1. None
    2. 1 hour or less
    3. 1-2 hours
    4. 2-4 hours
    5. 4-6 hours
    6. 6 or more hours
21. In the last month, approximately how much of the day do you typically spend watching or reading the news coverage about the outbreak through informal news sources (e.g., social media posts, blogs, talk shows; does not include links from social media to actual news source articles)?
    1. None
    2. 1 hour or less
    3. 1-2 hours
    4. 2-4 hours
    5. 4-6 hours
    6. 6 or more hours
22. Was your school closed as a result of the coronavirus?
    1. Yes
    2. No
23. [If 66a]: Approximate date:
24. [If 66a]: Are you continuing with your school work while you’re at home?
    1. Yes
    2. No
25. [If 68a]: How are you continuing to do your school work?
    1. My parent is homeschooling me
    2. My teachers sent home work
    3. I’m doing my classes online
26. [If 68a]: What is the environment like when you are doing your school work?
    1. Very quiet
    2. Somewhat quiet
    3. Somewhat noisy
    4. Very noisy
27. [If 68a]: How difficult is it to get school work done?
    1. Not at all difficult
    2. A little difficult
    3. Somewhat difficulty
    4. Very difficult
28. Have you been social distancing (e.g., putting at least 6 feet of physical distance between yourself and other people who do not live in your household)?
    1. Yes
    2. No
29. [If 72a]: For how long have you been social distancing?
    1. 1-2 days
    2. 2-4 days
    3. 4-7 days
    4. 1-2 weeks
    5. More than 2 weeks
30. On average, how much have you and your family followed social distancing recommendations?
    1. We have made no changes to our behavior.
    2. We have made minor changes to reduce physical social contact (e.g., going out less, seeing fewer friends)
    3. We have made major changes to reduce physical social contact (e.g., not going to school / work, limiting contact with people outside our home)
    4. Completely changed (e.g., staying inside almost all the time, only going out for necessities, and keeping my physical distance from other people when we do)
31. On average, how much have your peers followed social distancing recommendations?
    1. They have made no changes to their behavior.
    2. They have made minor changes to reduce physical social contact (e.g., going out less, seeing fewer friends)
    3. They have made major changes to reduce physical social contact (e.g., not going to school / work, limiting contact with people outside our home)
    4. Completely changed (e.g., staying inside almost all the time, only going out for necessities, and keeping their physical distance from other people when they do)
32. In the last month, what types of regular, organized group activities have you been participating in during or outside of school, ***even if remotely***? (Check all that apply)
    1. School clubs
    2. Sports / athletic activity (e.g., soccer, dance, running)
    3. Musical or artistic activities
    4. Other: [Fill]
    5. I do not participate in any
33. [If 76a]: How many days a week have you been participating in school clubs?
    1. Less than 1 day a week
    2. At least 1 day a week
    3. 2-3 days a week
    4. 4-5 days a week
    5. Every day or nearly every day
34. [If 76b]: How many days a week have you been participating in sports / athletic activity?
    1. Less than 1 day a week
    2. At least 1 day a week
    3. 2-3 days a week
    4. 4-5 days a week
    5. Every day or nearly every day
35. [If 76c]: How many days a week have you been participating in musical or artistic activities?
    1. Less than 1 day a week
    2. At least 1 day a week
    3. 2-3 days a week
    4. 4-5 days a week
    5. Every day or nearly every day
36. [If 76d]: How many days a week have you been participating in [other filled]?
    1. Less than 1 day a week
    2. At least 1 day a week
    3. 2-3 days a week
    4. 4-5 days a week
    5. Every day or nearly every day
37. How much do you miss participating in your normal activities?
    1. Not at all
    2. A little
    3. Somewhat
    4. Very
38. In the last month, how often have you seen your friends?
    1. Never
    2. Less than once a week
    3. Once a week
    4. 2-3 times a week
    5. Once a day
    6. Multiple times a day
39. How often have you been talking to friends on the phone?
    1. Never
    2. Less than once a week
    3. Once a week
    4. 2-3 times a week
    5. Once a day
    6. Multiple times a day
40. How often have you been texting friends?
    1. Never
    2. Less than once a week
    3. Once a week
    4. 2-3 times a week
    5. Once a day
    6. Multiple times a day
41. How often have you been talking to friends on other messaging apps (WhatsApp, etc.)?
    1. Never
    2. Less than once a week
    3. Once a week
    4. 2-3 times a week
    5. Once a day
    6. Multiple times a day
42. How often have you been talking to friends on social media (SnapChat, Instagram, Facebook, etc.)?
    1. Never
    2. Less than once a week
    3. Once a week
    4. 2-3 times a week
    5. Once a day
    6. Multiple times a day
43. In the last month, how often have you been seeing your parents?
    1. Never
    2. Less than once a week
    3. Once a week
    4. 2-3 times a week
    5. Once a day
    6. Multiple times a day
44. How often have you been talking to your parents on the phone?
    1. Never
    2. Less than once a week
    3. Once a week
    4. 2-3 times a week
    5. Once a day
    6. Multiple times a day
45. How often have you been texting with your parents?
    1. Never
    2. Less than once a week
    3. Once a week
    4. 2-3 times a week
    5. Once a day
    6. Multiple times a day
46. How often have you been talking to your parents on other messaging apps (WhatsApp, etc.)?
    1. Never
    2. Less than once a week
    3. Once a week
    4. 2-3 times a week
    5. Once a day
    6. Multiple times a day
47. How often have you been talking to your parents on social media (SnapChat, Instagram, Facebook, etc.)?
    1. Never
    2. Less than once a week
    3. Once a week
    4. 2-3 times a week
    5. Once a day
    6. Multiple times a day
48. In the last month, how often did a peer leave you out of an ***online/digital*** group activity or conversation that you really wanted to be included in (e.g., group chat, group photo, etc.)?
    1. Never
    2. Less than once a week
    3. Once a week
    4. 2-3 times a week
    5. Once a day
    6. Multiple times a day
49. How often did a peer threaten to hurt you or beat you up using an ***online/digital*** platform (texting, social media app, photo caption, etc.)?
    1. Never
    2. Less than once a week
    3. Once a week
    4. 2-3 times a week
    5. Once a day
    6. Multiple times a day
50. How often did a peer tease you in a mean way saying rude things or calling you bad names using an ***online/digital*** platform (texting, social media app, photo caption, etc.)?
    1. Never
    2. Less than once a week
    3. Once a week
    4. 2-3 times a week
    5. Once a day
    6. Multiple times a day
51. How often did a peer try to damage your social reputation using an ***online/digital*** platform (texting, social media app, photo caption, etc.)?
    1. Never
    2. Less than once a week
    3. Once a week
    4. 2-3 times a week
    5. Once a day
    6. Multiple times a day
52. How often did a peer stop talking to you through an ***online/digital*** platform (texting, social media app, etc.)?
    1. Never
    2. Less than once a week
    3. Once a week
    4. 2-3 times a week
    5. Once a day
    6. Multiple times a day
53. How often did a peer block you using ***online/digital*** platform (texting, social media app, etc.)?
    1. Never
    2. Less than once a week
    3. Once a week
    4. 2-3 times a week
    5. Once a day
    6. Multiple times a day
54. How many hours a day do you typically spend passively scrolling through social media?
    1. None
    2. 1 hour or less
    3. 1-2 hours
    4. 2-4 hours
    5. 4-6 hours
    6. 6 or more hours
55. How many hours a day do you typically spend passively browsing other non-news websites?
    1. None
    2. 1 hour or less
    3. 1-2 hours
    4. 2-4 hours
    5. 4-6 hours
    6. 6 or more hours
56. How many hours a day do you typically spend watching leisure movies or shows?
    1. None
    2. 1 hour or less
    3. 1-2 hours
    4. 2-4 hours
    5. 4-6 hours
    6. 6 or more hours
57. How many hours a day do you typically spend reading books or magazines for leisure?
    1. None
    2. 1 hour or less
    3. 1-2 hours
    4. 2-4 hours
    5. 4-6 hours
    6. 6 or more hours
58. In the last month, have you felt that you have an adult in your life you could turn to for emotional support?
    1. Yes
    2. No
59. [If 102a]: What’s their relationship to you (check all that apply)?
    1. Parent
    2. Other family member
    3. Family friend
    4. Neighbor
    5. Coach
    6. Teacher
    7. Other mentor: [Fill]
60. [If 102a]: Think about the person you’d turn to the most for emotional support, how often have you talked to them in the last month?
    1. Not at all
    2. Every few weeks
    3. At least once a week
    4. Multiple times a week
61. In the last month, how many hours a day have you been spending actively socializing either in person or digitally with people ***not*** in your household?
    1. None
    2. 1 hour or less
    3. 1-2 hours
    4. 2-4 hours
    5. 4-6 hours
    6. 6 or more hours
62. [If 105b-f]: Rank most commonly used methods of communication:
    1. Social media apps
    2. Texting
    3. Phone calls
    4. Video-chatting
    5. In-person
63. In the last month, how many hours a day have you been spending actively socializing either in person or digitally with people ***who are*** in your household?
    1. None
    2. 1 hour or less
    3. 1-2 hours
    4. 2-4 hours
    5. 4-6 hours
    6. 6 or more hours
64. [If 107b-f]: Rank most commonly used methods of communication:
    1. Social media apps
    2. Texting
    3. Phone calls
    4. Video-chatting
    5. In-person
65. In the last month, do you feel more or less connected to the following people:
    1. Close Friends
       1. Much less connected
       2. A little less connected
       3. The same
       4. A little more connected
       5. Much more connected
    2. Other friends
       1. Much less connected
       2. A little less connected
       3. The same
       4. A little more connected
       5. Much more connected
    3. Family in household
       1. Much less connected
       2. A little less connected
       3. The same
       4. A little more connected
       5. Much more connected
    4. Family not in household
       1. Much less connected
       2. A little less connected
       3. The same
       4. A little more connected
       5. Much more connected
    5. Other adult mentors
       1. Much less connected
       2. A little less connected
       3. The same
       4. A little more connected
       5. Much more connected
    6. Your community
       1. Much less connected
       2. A little less connected
       3. The same
       4. A little more connected
       5. Much more connected
66. In the last month, how much have you missed being with people ***who do not live with you:***
    1. Close Friends
       1. Not at all
       2. A little
       3. Somewhat
       4. Very
    2. Other friends
       1. Not at all
       2. A little
       3. Somewhat
       4. Very
    3. Family
       1. Not at all
       2. A little
       3. Somewhat
       4. Very
    4. Teachers
       1. Not at all
       2. A little
       3. Somewhat
       4. Very
    5. Community
       1. Not at all
       2. A little
       3. Somewhat
       4. Very
67. In the last month, how often have you felt lonely?
    1. Never
    2. Once
    3. Several Times
    4. A few times a week
    5. Nearly every day
68. In the last month, what have you typically been doing for physical exercise?
    1. Biking
    2. Running
    3. Dance
    4. Organized sport
    5. Swimming
    6. Yoga or pilates
    7. Aerobics or other cardio
    8. Other: [Fill in]
    9. I have not engaged in physical exercise
69. [If selected 112a-h]: On average, how often have you exercised?
    1. Less than 1 day a week
    2. At least 1 day a week
    3. 2-3 days a week
    4. 4-5 days a week
    5. Every day or nearly every day
70. [If selected 112a-h]: On average, how long have you typically exercised for?
    1. Less than 30 minutes
    2. 30-60 minutes
    3. 60-90 minutes
    4. 90+ minutes
71. In the last month, how often have you spent time in outdoors green spaces (e.g., open spaces including parks, canals, nature areas, coastal or beach front, countryside, farmland)?
    1. Less than 1 day a week
    2. At least 1 day a week
    3. 2-3 days a week
    4. 4-5 days a week
    5. Every day or nearly every day
72. How often have you spent time outside your home for at least 30 minutes not including outdoor green spaces listed above (e.g., back yard, neighborhood street)?
    1. Less than 1 day a week
    2. At least 1 day a week
    3. 2-3 days a week
    4. 4-5 days a week
    5. Every day or nearly every day
73. In the last month, how well have you been sleeping? My sleep quality has been:
    1. Not good
    2. Somewhat good
    3. Mostly good
    4. Very good
74. On an average night, how many hours did you sleep?
    1. [fill] hours
75. If you experienced trouble sleeping, was it because of (check all that apply or leave blank if no trouble sleeping):
    1. Trouble falling asleep
    2. Waking up during the night
    3. Waking up earlier than you wanted to
76. In the last month, have your days had a fairly consistent routine?
    1. Not at all, every day was different
    2. Somewhat, I did some things at the same time every day
    3. Mostly, I did most things at the same time every day
    4. Very much, I did everything at the same time every day
77. Have family members really helped and supported each other?
    1. Not at all
    2. Somewhat
    3. Mostly
    4. Very much
78. Has there been a feeling of togetherness in your family?
    1. Not at all
    2. Somewhat
    3. Mostly
    4. Very much
79. Have family members rarely criticized each other?
    1. Not at all
    2. Somewhat
    3. Mostly
    4. Very much
80. How have your parents been discussing the coronavirus outbreak?
    1. Not at all seriously (e.g., they say it’s not a big deal)
    2. A little seriously (e.g., they say we just have to be a little more careful)
    3. Somewhat seriously (e.g., they say we should change a lot of our behavior)
    4. Very seriously (e.g., they say that it’s very dangerous and it’s important to change our behavior)
81. Do you have a difficult relationship with someone who lives in your home? If so, who? Check all that apply.
    1. Yes- Parent
    2. Yes- Sibling
    3. Yes- Other [fill in]
    4. No
82. [If 125a-c]: Has being at home with this person more changed things in your relationship? [logic, appears for each relationship selected]
    1. Yes, gotten worse
    2. Yes, gotten better
    3. No, stayed about the same
83. [If 125a-c]: How upsetting is it to be at home more often with this person?
    1. Not at all upsetting
    2. A little upsetting
    3. Somewhat upsetting
    4. Very upsetting
84. Have you or anyone in your household experienced any racism, prejudice, or discrimination (i.e., being treated unfairly because of some aspect of your identity) related to the coronavirus outbreak?
    1. No
    2. Yes
       1. [if b selected] Racial slur
       2. [if b selected] Avoided because of my/their race or ethnicity
       3. [if b selected] Attacked because of my/their race or ethnicity
       4. [if b selected] Explain [Fill in]
85. Do you trust the government or leaders of your community to take the appropriate steps to manage your safety?
    1. Not at all
    2. A little
    3. Somewhat
    4. Very
86. How well do you feel like you understand the current pandemic and the reasoning behind social distancing?
    1. Not at all
    2. A little
    3. Somewhat
    4. Very
87. For each item below, indicate ***the most worried you have felt in the last month*** using the sliding scale from ‘not at all worried’ to ‘extremely worried.’

|  | **1** | **2** | **3** | **4** | **5** | **6** | **7** | **8** | **9** | **10** |
| --- | --- | --- | --- | --- | --- | --- | --- | --- | --- | --- |
| You would become seriously ill or die |  |  |  |  |  |  |  |  |  |  |
| One or both of your parents would become seriously ill or die |  |  |  |  |  |  |  |  |  |  |
| Someone else you care about would be become seriously ill or die |  |  |  |  |  |  |  |  |  |  |
| You would be unable to access care if you were sick |  |  |  |  |  |  |  |  |  |  |
| Someone you care about would be unable to access care if they got sick |  |  |  |  |  |  |  |  |  |  |
| Infected items would be brought into your home |  |  |  |  |  |  |  |  |  |  |
| Need to disinfect items brought into your home |  |  |  |  |  |  |  |  |  |  |
| Your family wouldn’t be able to pay bills |  |  |  |  |  |  |  |  |  |  |
| Your family wouldn’t have enough money for basic needs like food and shelter |  |  |  |  |  |  |  |  |  |  |
| Your family would lose their housing |  |  |  |  |  |  |  |  |  |  |
| Your parent(s) would lose their job(s) |  |  |  |  |  |  |  |  |  |  |
| Your family wouldn’t be able to get enough food |  |  |  |  |  |  |  |  |  |  |
| Your family wouldn’t be able to get other necessities like toilet paper and cleaning supplies |  |  |  |  |  |  |  |  |  |  |
| Your family wouldn’t be able to get medicine |  |  |  |  |  |  |  |  |  |  |
| Someone else that you care about wouldn’t be able to pay bills |  |  |  |  |  |  |  |  |  |  |
| Someone else that you care about wouldn’t have enough money for basic needs like food and shelter |  |  |  |  |  |  |  |  |  |  |
| Someone else that you care about would lose their housing |  |  |  |  |  |  |  |  |  |  |
| Someone else that you care about would lose their job(s) |  |  |  |  |  |  |  |  |  |  |
| Someone else that you care about wouldn’t be able to get enough food |  |  |  |  |  |  |  |  |  |  |
| Someone else that you care about wouldn’t be able to get other necessities like toilet paper and cleaning supplies |  |  |  |  |  |  |  |  |  |  |
| Someone else that you care about wouldn’t be able to get medicine |  |  |  |  |  |  |  |  |  |  |
| Something bad would happen if you went outside |  |  |  |  |  |  |  |  |  |  |
| That you wouldn’t do well in school |  |  |  |  |  |  |  |  |  |  |
| That you made someone else sick with the coronavirus |  |  |  |  |  |  |  |  |  |  |
| That you or someone you know would be the victim of racism or discrimination |  |  |  |  |  |  |  |  |  |  |
|  |  |  |  |  |  |  |  |  |  |  |
|  |  |  |  |  |  |  |  |  |  |  |

1. In the last month, how have you dealt with any stress or anxiety related to the coronavirus? (Check all that apply)
   1. Watched/read news
   2. Tried to distract yourself
   3. Thought about all the details of the problem
   4. Talked to family or friends
   5. Exercised
   6. Meditated
   7. Sought counseling from a therapist or religious leader
   8. Self-care activities
      1. Explain [Fill in]
   9. Other
      1. Explain [Fill in]
2. Have you done any of the following since the beginning of the outbreak?
   1. Volunteered time at hospitals
   2. Donated/prepared food
   3. Donated money/supplies
   4. Gave shelter to displaced people
   5. Prayed/prayer group/prayer vigil
   6. Wrote letters or contacted isolated older people
   7. Cheered on health care workers
   8. Other way of helping (specify _____________________)
3. Taking everything into consideration, what was the most stressful part of the coronavirus outbreak and subsequent social distancing/quarantine for you personally?
   1. [Fill in]
4. Is there anything we should know about the psychological effects of the outbreak that was not covered in this survey? If no, leave blank.
   1. [Fill in]
